# RNA recombination at Chikungunya virus 3'UTR as an evolutionary mechanism that provides adaptability

**DOI:** 10.1371/journal.ppat.1007706

**Published:** 2019-04-15

**Authors:** Claudia V. Filomatori, Eugenia S. Bardossy, Fernando Merwaiss, Yasutsugu Suzuki, Annabelle Henrion, María Carla Saleh, Diego E. Alvarez

**Affiliations:** 1 Instituto de Investigaciones Biotecnológicas, Universidad Nacional de San Martín, Buenos Aires, Argentina; 2 Institut Pasteur, Viruses and RNA Interference Unit, Centre National de la Recherche Scientifique UMR 3569, Paris, France; Colorado State University, UNITED STATES

## Abstract

The potential of RNA viruses to adapt to new environments relies on their ability to introduce changes in their genomes, which has resulted in the recent expansion of re-emergent viruses. Chikungunya virus is an important human pathogen transmitted by mosquitoes that, after 60 years of exclusive circulation in Asia and Africa, has rapidly spread in Europe and the Americas. Here, we examined the evolution of CHIKV in different hosts and uncovered host-specific requirements of the CHIKV 3’UTR. Sequence repeats are conserved at the CHIKV 3’UTR but vary in copy number among viral lineages. We found that these blocks of repeated sequences favor RNA recombination processes through copy-choice mechanism that acts concertedly with viral selection, determining the emergence of new viral variants. Functional analyses using a panel of mutant viruses indicated that opposite selective pressures in mosquito and mammalian cells impose a fitness cost during transmission that is alleviated by recombination guided by sequence repeats. Indeed, drastic changes in the frequency of viral variants with different numbers of repeats were detected during host switch. We propose that RNA recombination accelerates CHIKV adaptability, allowing the virus to overcome genetic bottlenecks within the mosquito host. These studies highlight the role of 3’UTR plasticity on CHIKV evolution, providing a new paradigm to explain the significance of sequence repetitions.

## Author summary

An emergent virus is a virus that has adapted to new hosts or environments through changes in its viral genome. Using as model chikungunya virus, which had explosively spread in the Americas and Europe during the last decade, we studied viral adaptability to mosquito and mammalian hosts. Natural isolates from recent outbreaks display highly different 3’UTRs, which result in different fitness in mosquito cells. Importantly, drastic changes in viral populations occur when the virus jumps between hosts, with different viral variants being positively and negatively selected according to fitness parameters. Frequent events of RNA recombination, in which the viral polymerase switches templates during RNA synthesis, were observed at the viral 3’UTR. RNA recombination explains the high plasticity of this part of the genome and the subsequent emergence of new viral variants that allows the virus to replicate in disparate systems. Our findings support a new model where viral diversity generated by RNA recombination is a potent viral strategy to overcome the fitness barriers imposed during host switching.

## Introduction

RNA viruses occur in genetically diverse populations that allow viruses to replicate under unfavorable conditions, evade immune responses, and alternate between different hosts [1–4]. Rapid adaptation depends on the generation and positive selection of beneficial changes; and relies mainly on two molecular mechanisms: point mutations and genomic recombination [5–9]. Point mutations arise from the introduction of nucleotide changes by viral RNA-dependent RNA-polymerases that lack proofreading activity. In turn, genomic recombination does not produce new changes at the nucleotide level. Instead, the rearrangement of existing genomes creates novel lineages and strains and it has resulted in re-emergence of many RNA viruses.

Chikungunya virus (CHIKV) is an arthropod borne virus that after 60 years of exclusive circulation in Asia and Africa has recently spread in Europe and America, producing about 1.7 million infections [10–14]. Explosive spread of CHIKV has been associated with adaptation of viral lineages to mosquito vector hosts that assured efficient transmission. For instance, the 2007 epidemics in La Reunion Island, which passed to the European continent, have been attributed to adaptive mutations within viral structural glycoproteins that allowed viruses to more readily infect the mosquito *Aedes albopictus* [15]. On the other hand, CHIKV strains that arrived to the Americas in December 2013 have been spread mainly by *Aedes aegypti* mosquitoes [16] and do not contain this adaptive mutation. Instead, they have acquired nucleotide modifications in the non-structural proteins ORF and sequence duplications at the 3’UTR [17].

CHIKV is an alphavirus in the Togaviridae family of viruses and contains a non-segmented single stranded RNA genome of 11–12 kb, with a type 0 cap at the 5’ end and a poly (A) tail at the 3’ end. The 3’UTR exhibits large size variations, including extensive substitutions, insertions and deletions, suggesting that it has evolved quickly, probably due to different evolutionary selective pressures. One interesting feature about CHIKV 3’UTR is that it contains short sequence repetitions named *direct repeats* (DRs) [18,19]. Notably, copy number of DRs varies among ECSA (East, Central, and South African) and Asian lineages, indicating that the 3’UTR diverged from a common ancestor that suffered historical events of duplication. Sequences of CHIKV DRs are relatively conserved between closely related lineages, indicating that they have functional significance [20]. The molecular bases that lead to CHIKV genetic diversity and to viral replacements in nature are not well understood. However, complex host-virus interactions could result in the transmission of only certain variants that exist in the viral population. In this regard, recent studies show that deletion of the repeated sequences reduces viral replication rate in mosquito cells but not in mammalian cells [17,21,22], suggesting that the DRs have host-specific functions. Nevertheless, the host selective pressures that act on these elements and their functional relevance in viral transmission are still poorly understood.

It is known that arbovirus populations experience important bottlenecks in their mosquito vectors, related to the anatomical barriers the virus has to cross within insects [23–25]. Environmental factors, mosquito species and subspecies, and the combinations of viruses and mosquito species affect bottleneck size. In some cases, bottlenecks during natural transmission could constrain genetic diversity of viral populations to levels that lead to fixation of less adapted viruses. So far, how genetic bottlenecks act in concert with host selective pressures to shape the composition of CHIKV populations remains unclear.

Sequence comparisons of RNA viruses from different groups show that those that must cycle between host species have a more diverse 3’UTR than others with a single host [26]. In this regard, we have previously introduced the concept that 3’UTR duplications of complex RNA structures provide mosquito-borne flaviviruses with the means to efficiently switch between hosts maintaining high viral fitness [27,28]. Here, based on the heterogeneity of the 3’UTR of circulating CHIKV, we hypothesized that the number of sequence repetitions can change to accelerate viral adaptation during continuous host cycling. We found that the 3’UTR of CHIKV populations is highly dynamic in cell culture and during the infection of laboratory mosquitoes, recapitulating the observations in natural isolates. Genetic diversity of viral populations depends both on CHIKV lineage and the infected host cell, indicating that CHIKV 3’UTR is under conflicting selective pressures in mosquito and mammalian cells. Our studies suggest that CHIKV uses genetic recombination at the 3’UTR to generate virus populations with diverse 3’UTRs. This strategy allows CHIKV to overcome bottlenecks in nature, driving the generation of new viral variants that rapidly replace the existing ones during adaptation to the host. Altogether, our findings provide important insights into the function of CHIKV 3’UTR plasticity for host cell adaptation, pointing out a central role of RNA recombination on CHIKV ongoing evolution.

## Results

### 3’UTR heterogeneity of re-emergent natural isolates

**Fig 1:**
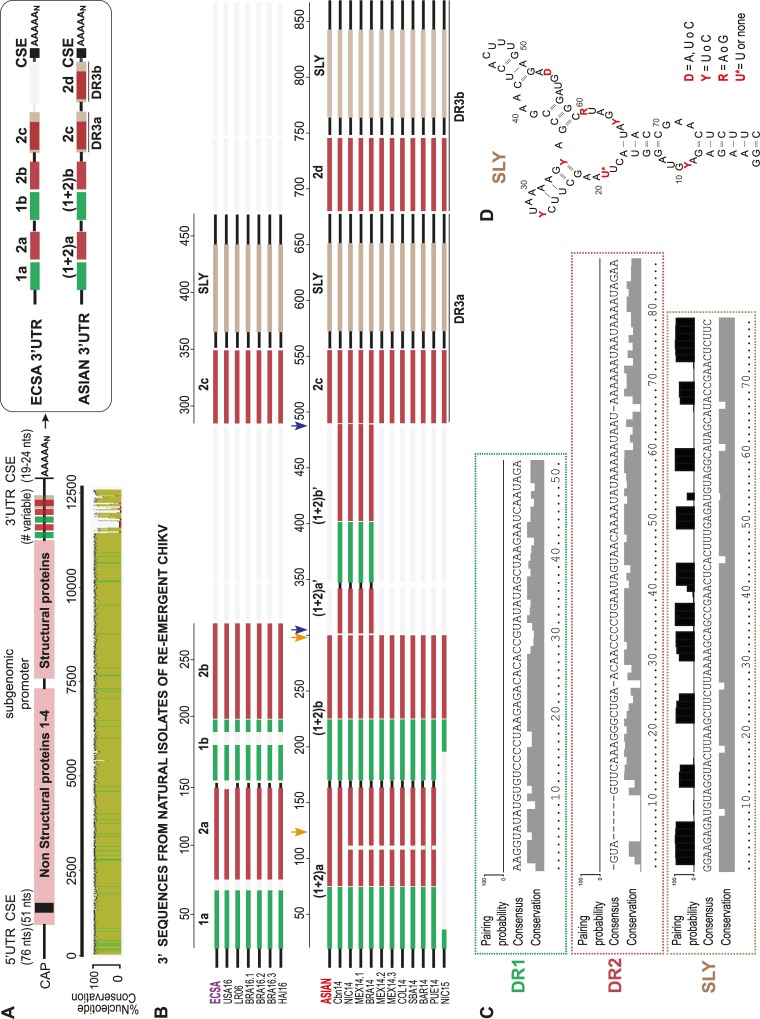
The 3’UTR of re-emerging CHIKV isolates. **(A)** Schematic representation (top) and nucleotide conservation plot (bottom) of the CHIKV complete genome and of the 3’UTR from the original ECSA and Asian strains (right). Direct Repeats (DRs) are represented with rectangular colored blocks and the Conserved Sequence Element (CSE) is shown in black. **(B)** Sequence alignment of re-emergent CHIKV. Alignment of ECSA- and Asian-derived re-emergent CHIKVs from 17 natural isolates, showing genetic variability in the number of DRs. Sequences are grouped by lineages. GeneBank accession numbers according to references are: HM045811.1 (ECSA), KY575567.1 (USA16), AM258994.1 (LR06), KY704954l (BRA16.1), KY704955 (BRA16.2), KY055011.1 (BRA16.3), MG000876.1 (HAI16), KT308163 (Asian), LN898111.1, (Cbn14), KY704000.17 (NIC15), KT327163.2 (MEX14.1), KP164571 (BRA14), KP851710.1 (MEX14.2), KP851709.1 (MEX14.3), KR559491 (COL14), KR55949 (SBA14), KY435464.1 (BAR14), KR264949.1 (PUE14), KY704001.17 (NIC15). Direct repeats are represented with colored lines and the absence of the respective sequences is shown with light grey lines. The 177-nt extra copy indicated between blue arrows in Cbn-CHIKV corresponds to the duplication of the sequence contained within orange arrows. **(C)** Base-pair probabilities, consensus sequences, and nucleotide conservation plots for DR1 (top), DR2 (middle) and SLY (bottom) at CHIKV 3’UTR. Sequence alignments were generated and analyzed using MAFFT and RNAalifold software, respectively. For ECSA-derived strains, DR1 corresponds to nucleotide positions 18–70 and 149–193; DR2 corresponds to 71–148, 194–265 and 281–349; and SLY corresponds to 364–442 in LR06 sequence. For Asian-derived strains DR1 corresponds to nucleotide positions 18–70, 168–223 and 345–400; DR2 corresponds to 81–160, 224–297, 401–486, 487–555 and 674–739; and SLY corresponds to 570–648 and 755–832 in Cbn14 sequence. **(D)** Predicted secondary RNA structure for SLY.

Three major CHIKV lineages have been defined based on their geographic distribution: West African, ECSA, and Asian [29,30]. ECSA and Asian strains have disseminated globally during the last decade, while the West African is confined to a sylvatic circulation in less populated areas of Africa. Interestingly, ECSA and Asian CHIKV lineages differ at their 3’UTRs. ECSA lineage contains two DR elements at the 3’UTR, namely DR1 (two copies) and DR2 (three copies), while the Asian 3’UTR accumulated insertions and point mutations around the DR1 and DR2 [hereafter designated as DR (1+2)], plus the duplication of an entire region (DR3) (Fig 1A) [21].

To study the 3’UTR diversity of contemporary re-emergent strains, we aligned and compared the 3’ sequences from 17 different natural isolates involved in recent CHIKV outbreaks (Fig 1B). In 2005, a CHIKV derived from ECSA lineage expanded the infection in La Reunion Island and then passed to the European continent. More recently, in 2016, a new autochthonous transmission of CHIKV derived from ECSA lineage was described in Brazil and Haiti [31–34]. ECSA-derived isolates contain simple 3’UTRs, with only two copies of DR 1 and DR2 (Fig 1B). In contrast, Asian-derived isolates, which arrived to America in 2013, contain longer 3’UTRs. Some American CHIKV isolates (such as the Caribbean) bear 3’UTRs that have never before been described in nature [17]. These novel 3’UTRs display a 177-nt duplication corresponding to the 3’ portion of the DR(1+2)a region plus an extra copy of the complete DR(1+2)b region (Fig 1B). It has been proposed that this duplication confers an advantage for viral replication in mosquito cells compared to the original Asian strain [17].

To explore whether sequence duplications are associated to the duplication of RNA secondary structures, we performed alignments of repeated sequence blocks and RNA folding predictions (Fig 1C and 1D). We found that DR1 and DR2 do not strictly fold into conserved structural elements, while the last portion of DR3 folds into a highly stable Y-shaped stem loop structure, which is supported by base pair probabilities and nucleotide conservation (Fig 1C) (21).

Altogether, Fig 1 shows that re-emergent CHIKV-isolates display different number of DR-copies at their 3’UTRs and that, despite certain nucleotide substitutions and deletions, many regions of the DRs are conserved between Asian and ECSA derived viruses. Importantly, CHIKV from different phylogenetic origins and bearing distinct 3’UTRs co-circulate in the same countries or nearby areas. This opens the question of how this complex mixture of variants will evolve in the near future to define the course of CHIKV epidemics.

### CHIKVs bearing Caribbean 3’UTR have an advantage to replicate in mosquito cells

**Fig 2:**
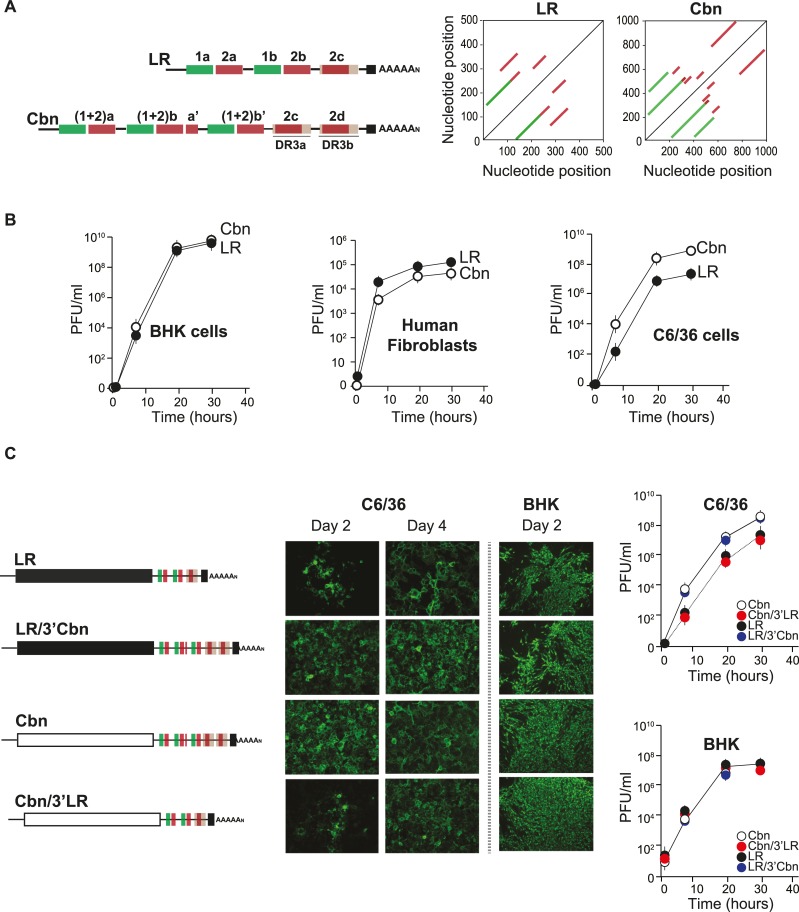
Replication kinetics of La Reunion (CHIKV-LR) and Caribbean (CHIKV-Cbn) lineages. **(A)** Left, schematic representation of the organization of the 3'UTRs of CHIKV-LR and CHIKV-Cbn. DRs are indicated with the same color code as in Fig 1. Right, RNA matrix comparison of CHIKV-LR and CHIKV-Cbn 3’UTRs, where each 3’UTR sequence was compared to itself, in order to search for repeated sequences patterns. Lines parallel to the central diagonal line evidence direct repeats. **(B)** Comparative growth kinetics of CHIKV-LR and CHIKV-Cbn in BHK, human fibroblasts, and C6/36 cells. Cells were infected with MOI = 0.1. Infectious viral particles were titrated by plaque assays at the indicated time points. Error bars represent standard deviations from the mean, n = 3. Data were analyzed by two-way ANOVA with Bonferroni´s test using PRISM5 (GraphPad Software). At 20h and 30h, p<0.01 in C6/36 and p<0.05 in human fibroblasts. **(C)** Expression of CHIKV proteins in C6/36 and BHK cells transfected with RNAs from CHIKV-LR and a chimeric CHIKV-LR carrying the Cbn-3’UTR (LR/3’Cbn), or the CHIKV-Cbn and a chimeric CHIKV-Cbn carrying LR-3’UTR (Cbn/3’LR). Viral replication was monitored by immunofluorescence assay as a function of time after RNA transfection using specific CHIKV antibodies (left) and virus growth curves (right). Error bars represent standard deviations from the mean, n = 3. Data were analyzed by two-way ANOVA with Bonferroni´s test using PRISM5 (GraphPad Software). At 20h and 30h, p<0.05 for Cbn/3’LR compared to the Cbn, and for LR/3’Cbn compared to LR.

Ongoing CHIKV epidemics are caused by viruses from ECSA and Asian lineages, which contain differences both in their coding and non-coding regions. As mentioned, the CHIKV responsible for 2005–2006 epidemics in La Reunion Island (CHIKV-LR; ECSA-derived) contained a single mutation in the envelope protein gene (E1-A226V) that increases viral fitness in *Aedes albopictus* [15,35], which is not present in the CHIKV that arrived to America [CHIKV-Caribbean (Cbn); Asian-derived]. Besides, both linages largely differ at their 3’UTRs. To identify sequence repetitions at the 3’UTR from these two distinct re-emergent viruses, RNA matrix comparisons were performed. Local alignment plots confirm the presence of multiple DRs at both 3’UTRs, but with distinct repetition patterns (Fig 2A). Firstly, we studied the impact of distinct 3’UTRs on replication by comparing growth kinetics of CHIKV-LR and CHIKV-Cbn in mammalian (BHK and human fibroblasts) and mosquito cells. Growth curves in mammalian cells showed that both viruses replicated similarly, despite a slight advantage of CHIKV-LR over the CHIKV-Cbn for replication in human fibroblasts (Fig 2B). In contrast, CHIKV-Cbn exhibited enhanced growth kinetics in *Aedes albopictus* C6/36 cells, resulting in 10-fold higher titers compared to CHIKV-LR. To assess the contribution of the 3’UTR to the differential behavior in insect cells, we designed chimeras containing the 3’UTR from CHIKV-LR in the context of the CHIKV-Cbn genome and vice versa. Equal amounts of RNA transcripts from parental and chimeric viruses were individually transfected into BHK or C6/36 cells. Then, virus replication was monitored by immunofluorescence as a function of time and virus growth curves (Fig 2C). In BHK cells, similar levels of replication were observed for parental and chimeric viruses of both LR and Cbn lineages, demonstrating that neither the virus backbone nor the 3’UTR play lineage specific functions and that the 3’UTR may be swapped between viral isolates. On the other hand, in mosquito cells the 3’UTR from CHIKV-Cbn provided CHIKV-LR a net advantage for replication, displaying increased percentage of infected cells at two days after transfection and enhanced growth kinetics compared to the parental virus (Fig 2C). In contrast, the 3’UTR from CHIKV-LR disfavored replication of CHIKV-Cbn in the same cell line (Fig 2C). So far, our results indicate that CHIKV-Cbn contains a 3’UTR advantageous for viral replication in C6/36 cells but without benefit for replication in different mammalian cells.

### Diversity of CHIKV-Caribbean and CHIKV-La Reunion populations grown in mammalian or mosquito cells

**Fig 3:**
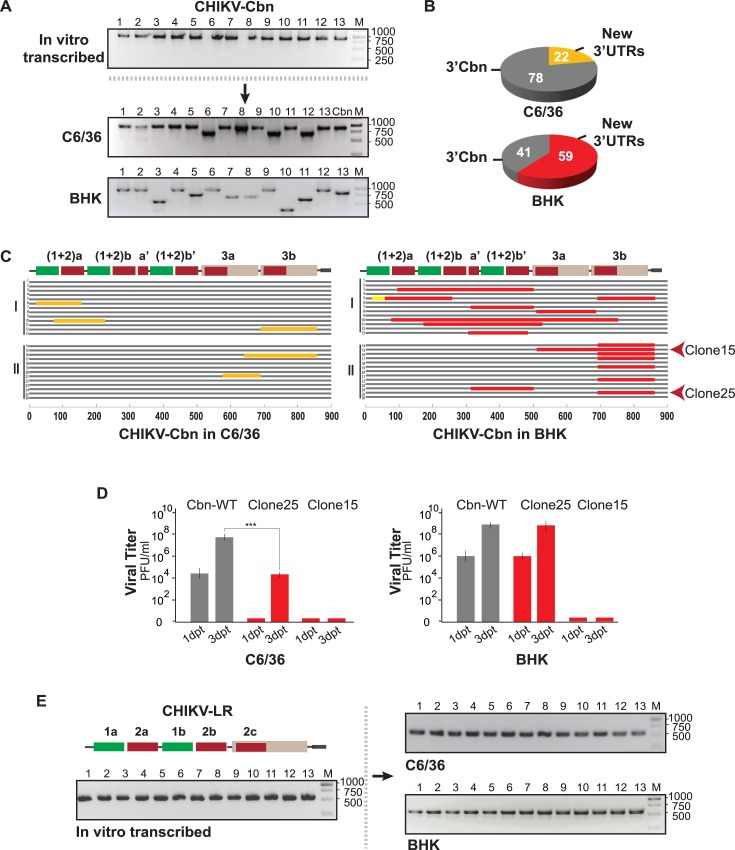
Composition of CHIKV-Cbn and CHIKV-LR populations grown in mosquito or mammalian cells. **(A)** Analysis of the 3'UTR of Caribbean lineage restricted to replicate in mosquito or mammalian cells. RNA was extracted from culture media and used for reverse transcription reaction. The pool of cDNA was amplified by PCR and cloned into pGEM-T Easy vector and individual clones were analyzed. Representative agarose gels for PCR amplification of the 3'UTRs of individual clones recovered from the input in vitro transcribed RNA (upper gel), and from viral populations after five successive passages in C6/36 (middle gel) and BHK (bottom gel) cells. Full-length 3'UTR of CHIKV-Cbn (Cbn) and DNA ladder (M) were used as reference. The sizes of DNA bands in the ladder (in base pairs) are indicated on the right. **(B)** Pie charts for the frequencies of parental 3'UTR (3'Cbn, grey) and viral variants with novel 3’UTRs, collectively referred to as New 3’UTRs (indicated in orange for C6/36 and in red for BHK cells). Mean percentages from experiments I and II are indicated in white. **(C)** Schematic representation of the alignment of sequences of CHIKV-Cbn populations grown in C6/36 (left) and BHK (right) cells from two independent experiments (I and II). Deletions within viral variants are indicated with orange and red lines. An 80-nt insertion in Clone 5 from mammalian-grown population is indicated with a yellow line. **(D)** Titers of infectious particles in the supernatant of mosquito (C6/36, left) and mammalian (BHK, right) cells after the transfection of CHIKV RNAs bearing the 3’UTRs of two viral variants obtained after passaging in mammalian cells. Clone 25 carried the deletion of a DR3 copy and was the most abundant viral variant from experiment II. Clone 15 had a drastic deletion of both DR3 copies. Data were analyzed by two-way ANOVA with Bonferroni´s test using PRISM5 (GraphPad Software). ***, p<0.001. **(E)** Analysis of the 3'UTR of La Reunion lineage restricted to replicate in mosquito or mammalian cells. Representative agarose gels for PCR amplification of the 3'UTRs of individual clones recovered from the input in vitro transcribed RNA (left gel), and from viral populations passaged in C6/36 (right upper gel) and BHK (right bottom gel) cells. DNA ladder (M) was used as reference. The sizes of DNA bands in the ladder (in base pairs) are indicated on the right.

Our observations regarding the differences in the replication of CHIKV bearing the 3’UTR of LR or Cbn lineages raise the questions of why does a less advantageous 3'UTR become fixed and how is diversity in the 3'UTR generated. To address these questions, we investigated whether viral populations of re-emerging CHIKV are prone to change during passaging in cell culture. We first followed the evolution of the 3’UTR of viruses restricted to replicate in mammalian or mosquito cells. Viral RNA molecules were obtained by in vitro transcription from CHIKV-Cbn infectious clone and used to transfect C6/36 and BHK cell lines in two parallel experiments (Fig 3A). Viruses recovered from the transfection supernatants were serially passaged in mosquito or mammalian cells. Then, total RNA was extracted from culture supernatants and used as a template for reverse transcription reactions with an oligo(dT) primer. The pool of viral cDNAs was used to amplify fragments corresponding to the 3’UTR ends and then cloned into pGEM-T Easy vector. Individual plasmid clones were analyzed by agarose gel electrophoresis and sequenced using Sanger method. Also, the 3’UTR from in vitro transcribed input RNA was RT-PCR amplified, cloned, and used as reference.

As expected, the size of the 3’UTR clones generated from in vitro transcribed RNA was uniform, and corresponded to the full-length Cbn 3’UTR (Fig 3A, upper gel). Unlike the 3’UTR of input RNA, the frequency of full-length Cbn 3’UTR changed from 100% to 78% and different viral variants with shorter 3’UTRs emerged in mosquito cells (Fig 3A, middle gel; Fig 3B, upper chart). Interestingly, the CHIKV-Cbn grown in mammalian cells showed a more drastic change in the composition of viral population (Fig 3A, bottom gel). A reduction of the input variant frequency from 100% to 41% and the emergence of new 3’UTRs with a frequency of about 60% were observed (Fig 3B, bottom chart). Sequencing of 3’UTR variants revealed that they contained deletions of DR regions located at different positions of the 3’UTR (Fig 3C, 13 clones for each sample are shown). For instance, in mosquito cell passaged viruses we found a clean deletion of DR(1+2)a in Clone 6 and a deletion of DR3b in Clone 12. In mammalian cells, viral variants with different deletions throughout the 3’UTR appeared in the first experiment. In the second experiment, the majority of the variants isolated carried the same deletion, corresponding to one copy of DR3. In addition to deletion variants, a viral variant with an 80-nt insertion emerged in cell culture (clone 5). Nucleotide sequences of variants are shown in S1 and S2 Figs.

Our results indicate that CHIKV-Cbn 3’UTR is prone to lose DR copies in cell culture. Strikingly, passaging of the virus results in clean deletions of DR copies that arise with higher frequency in BHK cells, suggesting that replication in mammalian cells is the main source of genetic variability at the 3’UTR.

To unequivocally address the effect of new 3'UTRs on virus replication, we introduced into the parental CHIKV-Cbn backbone one of the 3’UTRs that was isolated with high frequency (Clone 25) and another with a drastic deletion (Clone 15). Then, we followed the production of infectious particles in both mammalian and mosquito cells. Clone 25, which carried the deletion of DR3a, showed delayed growth kinetics in mosquito cells while it did not affect viral replication in mammalian cells. Instead, Clone 15, which carried the complete deletion of both DR3 copies, was not able to replicate neither in mammalian nor mosquito cells (Fig 3D). Therefore, it is feasible that this variant represented the non-replicative RNA of a defective interfering particle. On the other hand, some of the variants obtained from mammalian cells bear deletions that delay viral replication solely in mosquito cells, posing a constraint to host-switch.

Contrarily from CHIKV-Cbn, CHIKV-LR bears the simplest 3’UTR found in nature. Then, we asked whether the 3’UTR of this lineage would also lose DR copies during passaging in cell culture. To this end, we similarly analyzed the 3’UTR from CHIKV-LR passaged populations. In contrast to our observations with CHIKV-Cbn, all the cloned fragments corresponded in size to the parental 3'UTR, for viruses grown in either mammalian or mosquito cells (Fig 3E). This result indicates that LR 3’UTR is much more stable than Cbn 3’UTR.

Altogether, these experiments show that CHIKV lineages have different potential to generate genetic variability at their 3’UTRs. While the CHIKV-Cbn (bearing multiple copies of DR elements at the 3’UTR) is greatly dynamic during single-host passaging, CHIKV-LR (with fewer copies) is seemingly stable in cell culture.

### New viral 3’UTR variants are generated by copy-choice recombination

**Fig 4:**
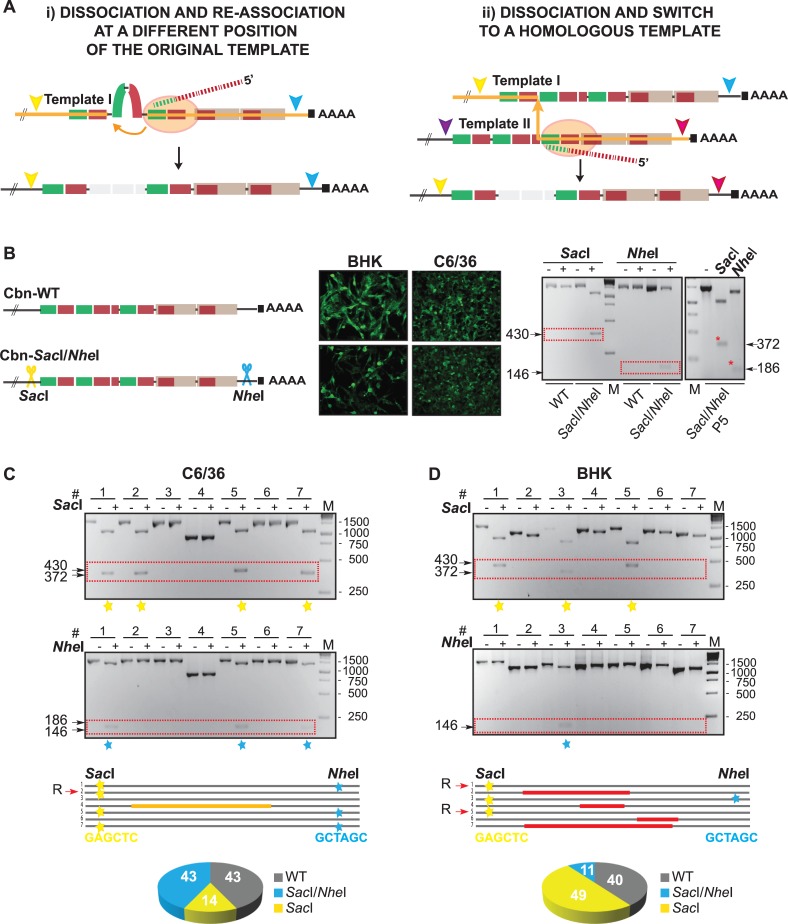
RNA recombination by switching of viral polymerase between homologous template strands. **(A)** Schematic representation of copy-choice recombination mechanisms, where the viral RNA-dependent RNA-polymerase (orange oval) and the nascent strand (dashed lines) jump at a different position of the original template (i) or between two homologous templates (ii). Orange lines indicate the possible route of viral polymerase. **(B)** To mark the CHIKV-Cbn RNA, *Sac*I and *Nhe*I restriction sites were introduced at the 5’ and 3’ ends of the viral 3’UTR, respectively. Immunofluorescence of Cbn-WT and Cbn-*Sac*I*/Nhe*I viruses were performed in BHK and C6/36 cells. Viral RNA was recovered from the supernatant, and fragments corresponding to the last ~1,500 nucleotides of the viral genome were obtained by RT-PCR, digested with *Sac*I and *Nhe*I restriction enzymes and analyzed by agarose gel electrophoresis. Undigested (-) and digested (+) products are shown on the right panel for parental (WT) and marked (*Sac*I/*Nhe*I) viruses. Also, we show the digested products for one representative individual clone of *Sac*I/*Nhe*I virus containing full-length 3’UTR, which was isolated after 5 passages in C6/36 cells (red asterisks). For P5, 20 clones were tested and they all conserved both *Sac*I and *Nhe*I restriction sites. **(C and D)** Cbn-WT and Cbn-*SacI/NheI* RNAs were co-transfected at a 1:1 ratio into C6/36 and BHK cells, in duplicates. At 72hpt, supernatants were harvested, subjected to RT-PCR and blunt-end cloned. For each experiment, 14 individual clones were subjected to PCR reactions, enzymatic digestion, and resolved by agarose gel electrophoresis. To illustrate, undigested and digested products from 7 representative clones are shown. Depending on the orientation of each inserted fragment into the blunt plasmid, digestion of *Sac*I positive clones generated 430 or 372 nucleotide-length products and digestion of *Nhe*I positive clones generated 186 or 146 nucleotide-length products. Presence of *SacI* and/or *NheI* restriction sites are indicated with yellow and light blue stars, respectively. Individual clones are also schematized and deletions are indicated with orange and red lines. Recombinant clones generated by template switching between strands are indicated with a R. Relative abundance of each virus is represented in pie charts (bottom). Unmarked WT 3'UTRs are indicated in grey, marked 3'UTRs sensitive to *Sac*I and *Nhe*I digestion in light blue, and 3'UTRs that are only sensitive to *Sac*I digestion in yellow.

Viral variants carrying deletions at their 3’UTR appeared in CHIKV populations. However, little is known about the molecular mechanism responsible for the generation of these new viral variants. It is accepted that RNA recombination contributes to genetic diversity of many RNA viruses, impacting on their evolution, epidemiology and re-emergence [6,36]. RNA deletions are often generated during RNA recombination by a copy-choice mechanism. This process involves dissociation of the RNA-dependent RNA-polymerase and the nascent strand from the RNA template, and their re-association at a different position of either the original RNA or template switch to a homologous RNA, guided by sequence similarities (Fig 4A, i and ii, respectively). Template switching from a donor to an acceptor strand generates a chimeric RNA molecule. Given the fact that CHIKV contains various copies of sequence repetitions at its 3’UTR, we reasoned that 3’ variability could be originated by a copy-choice mechanism. To differentiate between re-association on the same or an alternative template molecule, we introduced point mutations into CHIKV-Cbn infectious clone in order to generate recognition sites for two unique restriction enzymes at the boundaries of the 3’UTR. Thus, when the RNA marked with the restriction sites is mixed with parental unmarked RNA, recombination between homologous RNAs by template switching will generate a chimeric 3’UTR carrying only one of the marker sites. We engineered *Sac*I and *Nhe*I restriction sites at positions 1–6 and 858–863 after the translation stop codon. The recombinant CHIKV-Cbn *Sac*I*/Nhe*I was infective in both mammalian and mosquito cells (Fig 4B). To visualize the presence of *Sac*I and *Nhe*I sites, fragments corresponding to the last 1,500 nucleotides of the viral genome were obtained by PCR and separately digested with the two restriction enzymes (S3 Fig). The fragment amplified from the recombinant virus was sensitive to both *Sac*I and *Nhe*I digestion, and the introduced mutations were stable in cell culture even after 5 successive passages (Fig 4B, right panels). Then, to evaluate template-switching events, equal amounts of RNA transcripts for the parental and CHIKV-Cbn *Sac*I*/Nhe*I were co-transfected into cultured cells. At day 3 after transfection, total RNA was extracted from culture media, used as a template for reverse transcription reactions and cloned. The presence of *Sac*I and *Nhe*I sites was assessed by enzymatic digestion of PCR products amplified from individual clones. For confirmation, the 3’UTRs were also sequenced and relative abundances of viral variants were calculated. About 15% and 50% of the viruses recovered from mosquito and mammalian cells, respectively, contained full-length 3’UTRs with only one of the two restriction sites (Fig 4C and 4D). The emergence of these new recombinant variants, that were absent in the input mixture of RNAs, strongly suggests that they were products of a template switching mechanism generated by a polymerase that jumped from a RNA to the same position of a homologous RNA.

Unexpectedly, all recombinants contained only the *Sac*I site. We speculate that nucleotide changes introduced to generate the *Nhe*I recognition sequence impaired recombination at this site. Furthermore, deletion variants were generated with a frequency of 14% in C6/36 and 49% in BHK (compare to Fig 3), and they did not carry restriction site marks. We reasoned that they were generated either by dissociation of viral polymerase and re-association at a different position of the original template (Fig 4A i) or by template switching between two unmarked RNAs (Fig 4A ii).

Overall, these results are the first evidence that RNA recombination occurs at the 3’UTR of CHIKV by means of template switching driven by sequence homology of DRs, and provide a molecular mechanism for the generation of viruses with deletions in the DR regions. Despite differences in frequency, RNA recombination was observed in both mammalian and mosquito cells, suggesting that it is a general mechanism of genome variability.

### Deletion analysis reveals distinct roles of 3’UTR DRs in mammalian and mosquito cells

**Fig 5:**
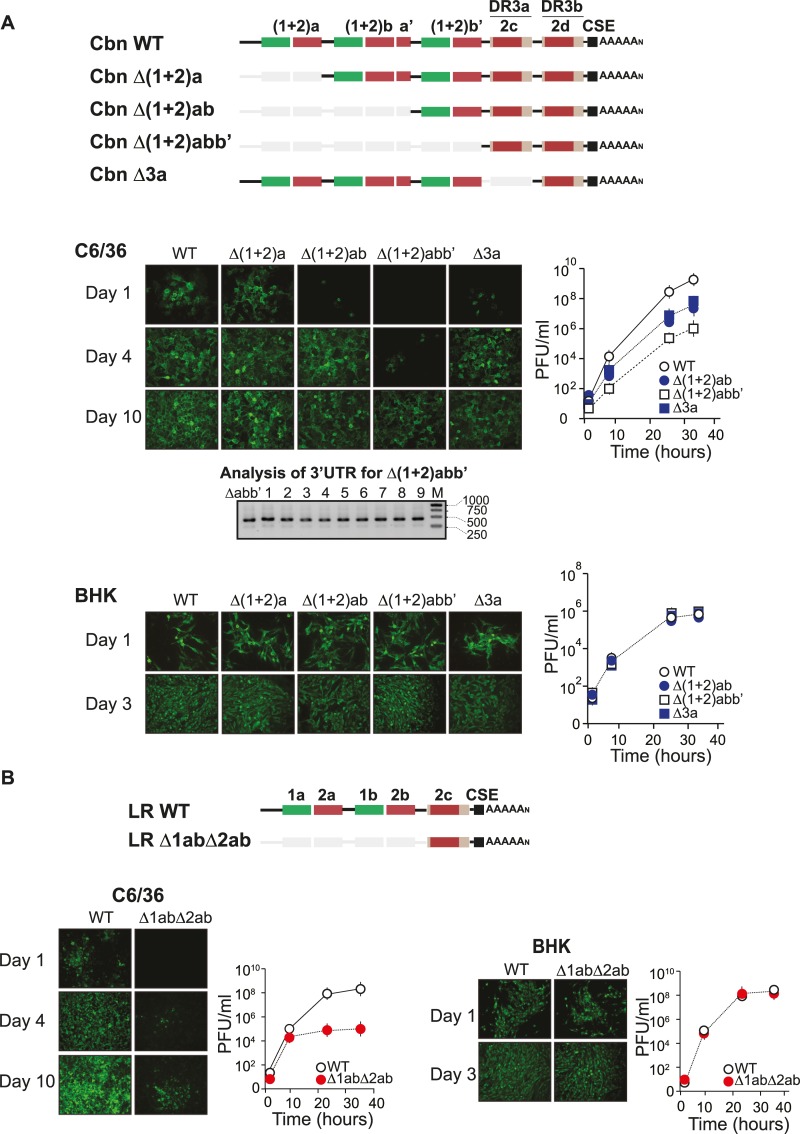
Requirements of DRs for CHIKV-Cbn and CHIKV-LR replication in mosquito and mammalian cells. **(A)** Top, schematic representation of the 3’UTR corresponding to recombinant CHIKV-Cbn. DR copies are represented with colored blocks and DR deletions are indicated with light grey blocks. Middle and bottom, immunofluorescence (left) and growth kinetics (right) of recombinant viruses compared to the parental CHIKV-Cbn (WT) performed in C6/36 cells and BHK cells, respectively. Error bars represent standard deviations from the mean, n = 3. Data were analyzed by two-way ANOVA with Bonferroni´s test using PRISM5 (GraphPad Software). Compared to the WT: p<0.001 for Δ(1+2)abb’ at 24h and 32h, Δ(1+2)ab at 24h and 32h, Δ3a at 24h; and p<0.05 for Δ3a at 32h. A representative agarose gel for PCR amplification of the 3'UTRs of individual clones is shown for Cbn Δ(1+2)abb’ population after 10 days post-transfection in C6/36 cells. DNA ladder (M) was used as reference. The sizes of DNA bands in the ladder (in base pairs) are indicated on the right. **(B)** Top, schematic representation of the 3’UTR corresponding to recombinant CHIKV-LR. DR copies are represented with colored blocks and DR deletions are indicated with light grey blocks. Left and right panels, immunofluorescence and growth kinetics of a recombinant virus carrying the deletion of both DR1 and DR2 copies (Δ1abΔ2ab) compared with the parental CHIKV-LR (WT) performed in C6/36 cells and BHK cells, respectively. Titer values are the mean +/- SD, n = 3. Data were analyzed by two-way ANOVA with Bonferroni´s test using PRISM5 (GraphPad Software). Compared to the WT, p<0.001 for Δ1abΔ2ab at 24h and 32h.

Even though we found that CHIKV is prone to loose DR copies by genome recombination, conservation of replicated DR copies is a common feature of alphavirus 3’UTRs, suggesting a functional role for duplication of DRs in CHIKV replication. To examine the requirements of DRs for replication in mammalian and mosquito cells we have deleted one, two or three copies of DR (1+2) or one DR3 copy from the CHIKV-Cbn infectious clone [Δ(1+2)a, Δ(1+2)ab, Δ(1+2)abb’, Δ3a respectively; Fig 5A]. Equal amounts of RNA transcripts for WT and mutant genomes were transfected into cells, and replication was followed by immunofluorescence staining of viral antigens as a function of time. We observed a direct association between the number of DRs and viral replication in mosquito cells. While deletion of one DR(1+2) copy had no effect compared to the WT, deletion of two or three DR(1+2) copies resulted in a moderate and a clear disadvantage for replication in mosquito cells, respectively (Fig 5A). One day post-transfection, the number of CHIKV positive cells were 33%, 35%, 5% and 0% for WT, Δ(1+2)a, Δ(1+2)ab and Δ(1+2)abb’, respectively. By day 4 post-transfection, the complete cell monolayer was infected with all viruses except for Δ(1+2)abb’; which only infected 10% of the cells. Because replication of Δ(1+2)abb’ virus was only evident after 10 days, we asked whether the virus recovered at this time showed any evidence of recombination at the 3’UTR. Analysis of clones corresponding to the Δ(1+2)abb’ population showed that the 3’UTR remained stable, suggesting that a minimum of DR copies may be maintained to favor recombination. In agreement with the immunofluorescence results, assessment of virus replication by growth curves showed that Δ(1+2)ab and Δ(1+2)abb’ exhibited delayed replication kinetics, resulting in 300 and 10,000 fold lower titers as compared to the WT, respectively (Fig 5A). Deletion of one DR3 copy was also evaluated in mosquito cells and it was found to delay viral replication, with 10% of the monolayer infected at day 1 and 100-fold reduction in viral titer compared to the WT. Replication of all mutants and WT viruses was similarly investigated in mammalian cells and interestingly, deletion of DR copies had no significant effect on viral fitness (Fig 5A, bottom panel; and S4 Fig). Taken together, our results indicate a remarkable difference for replication requirements in mosquito and mammalian cells, with an evident positive correlation between the number of DR copies and replication rates in mosquito cells. In addition, DR(1+2) copies cannot compensate for the deletion of DR3, suggesting that DR(1+2) and DR3 copies are functionally different. In fact, DR1 and DR2 do not fold into stable RNA structures. Because DR3 folds into a stable SLY (Fig 1D), it is tempting to speculate that this structure plays a role on viral replication in mosquito cells.

Next, we analyzed the importance of the DRs in the context of CHIKV-LR, deleting the two copies of DR1 and the two copies of DR2. In accordance with our previous results and with the observations by Morley et al. [22], the deletion caused a dramatic delay on viral replication in C6/36 cells, while it did not affect replication in BHK cells (Fig 5B).

In conclusion, deletion of DR copies had no effect on viral replication in mammalian cells, indicating that they are redundant in this host. In turn, in mosquito cells we found a positive correlation between the number of DRs and the replication rate.

### Dynamics of mammalian-adapted CHIKV populations during host switch

**Fig 6:**
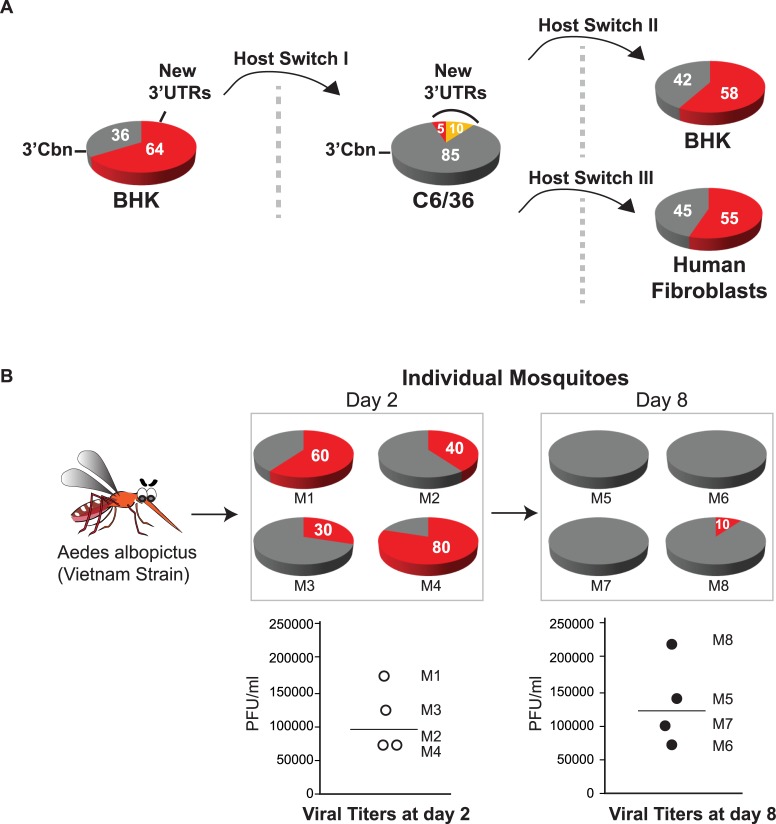
Dynamics of the 3’UTR of viral populations during host switch. **(A)** CHIKV-Cbn grown in mammalian cells (Fig 3, Experiment II) was used to infect C6/36 cells (Host Switch I; n = 2). Then, viral population released from mosquito cells was switched back to replicate in BHK cells (Host Switch II) or human fibroblasts (Host Switch III). Pie charts with frequencies of viral variants are shown (3’Cbn, grey and New 3’UTRs, red). Viral variants with New 3’UTRs after switching to mosquito cells are indicated in orange. **(B)** Oral infection of *Aedes albopictus* mosquitoes with CHIKV-Cbn grown in mammalian cells. On days 2 and 8, individual mosquitoes were collected for RNA extraction, 3’UTR analysis and virus titration. Pie charts with frequencies of 3’UTR variants (3’Cbn, grey and New 3’UTRs, red) obtained for each mosquito are shown. Virus titers for individual mosquitoes are shown. Lines represent the medians.

CHIKV populations are subjected to diversification during host cell adaptation. Since redundant DR copies do not provide an advantage for viral replication in mammalian cells, selection of viruses that rapidly loose DR copies is favored. On the other hand, duplication of DRs enhances replication in mosquito cells and thus, there is a positive selection pressure on DRs that explains the lower frequency of deletion variants in this host. We reasoned that host switch by alternate passaging of the virus in mammalian and mosquito cells would impact on the frequency of deletion variants. The CHIKV-Cbn grown in mammalian cells, where 64% of the viruses bear deletions in the 3’UTR was used to infect mosquito cells (Host Switch I, Fig 6A). Then, the 3'UTR of individual clones was analyzed as described previously (see Fig 3). As expected, the 3’UTR from the viral progeny released from mosquito cells showed a drastic change in the composition of viral population, with a prevailing of parental CHIKV-Cbn at a frequency of 85%. In addition, the viral variants that had arisen during adaptation to mammalian cells (Experiment II in Fig 3C) changed their frequency from 64% to 5%, and new mosquito-adapted variants emerged at a frequency of 10% (in orange, Fig 6A). Interestingly, when this mosquito-grown population was switched back to mammalian cells such as BHK or human fibroblasts (Host Switch II and III, Fig 6A), the deletion variants became nearly 60% of the population.

Additionally, we used the mammalian-grown population to infect *Aedes albopictus* mosquitoes. Two and eight days after infectious blood meal, viral titers were estimated and the frequency of viral variants in individual mosquitoes was assessed by analyzing 20 clones of the 3’UTR for each mosquito. Virus titers confirmed that the 8 mosquitoes have been infected (Fig 6B, bottom) and analysis of the 3’UTR sequences revealed that the composition of the input population changed within infected mosquitoes. While deletion variants represented 30 to 80% of the population at day 2, they were drastically outcompeted by viruses carrying full-length 3’UTRs by day 8 (Fig 6B, top). These results show that viral populations are dynamic when the virus shuttles from mammalian cells to mosquitoes, with different viral variants being negatively or positively selected in the host.

Collectively, these findings indicate that CHIKV 3’UTR is a major determinant for rapid adaptation to host switching. DRs are redundant in mammalian cells and thus, deletion mutants generated by RNA recombination are rapidly selected. In turn, because there is a requirement for DR duplication in mosquito cells, negative selection of deletion mutants occurs.

### RNA recombination occurs between the 3’UTR of different CHIKV lineages

We demonstrated that emergence of viral variants in mammalian cells by RNA recombination results in a significant fitness cost in mosquito cells, posing a constraint during host switching. Then, we asked whether positive selection and RNA recombination act concertedly to rescue viral replication in mosquito cells. Given the fact that variants of CHIKV carrying different but relatively conserved 3’UTRs co-circulate in the same geographic areas (see Fig 1), we hypothesized that RNA recombination between lineages could occur to generate chimeric variants with enhanced viral replication in mosquito cells. To investigate this possibility, we mixed CHIKV-LR with the chimeric virus containing the 3’UTR from CHIKV-Cbn in a 9:1 ratio in order to give a quantitative advantage to the virus with the shorter 3’UTR, and grew viral populations in C6/36 cells.

**Fig 7:**
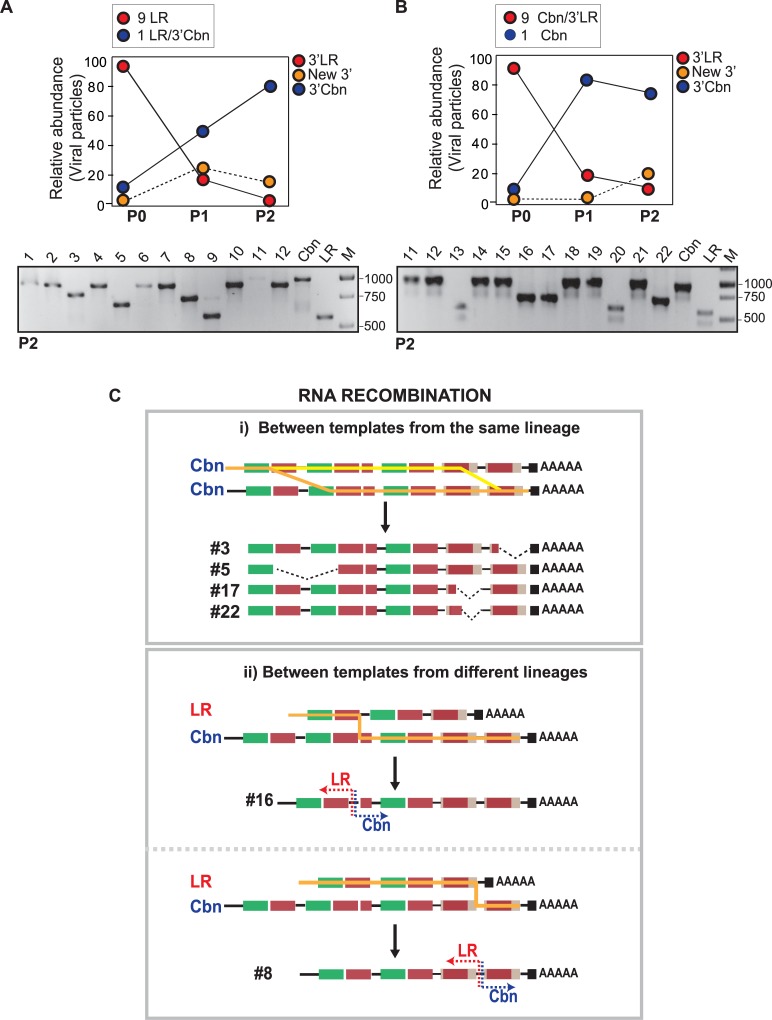
Co-transfection of viral RNAs containing the 3’UTR from CHIKV-Cbn and CHIKV-LR. **(A)** C6/36 cells were transfected with a mix of LR : LR/3’Cbn RNAs at a 9:1 ratio or **(B)** a mix of Cbn : Cbn/3’LR RNAs at a 1:9 ratio (P0). At 72 hpt, viruses were recovered from culture media (P1) and used to infect fresh C6/36 cells (P2, virus recovered from the second viral passage). Total RNA was purified from P0, P1 and P2, subjected to RT-PCR, cloning and sequencing. Agarose gels with PCR products from P2 individual clones are shown on the bottom. Amplicons from full-length 3'UTR of CHIKV-Cbn (Cbn), full-length 3'UTR of CHIKV-LR (LR), and a DNA ladder (M) are shown as reference. The size of DNA ladder (in base pairs) is indicated on the right. **(C)** Representation of the 3’UTRs of input RNAs and RNA products of recombination. Template switching events between strands from the same (Cbn & Cbn) or different (LR & Cbn) lineages are schematized. Orange lines indicate the possible route of viral polymerase. Numbers refer to the individual recombinant clones in panel (A) isolated from co-transfection experiment.

As expected, adaptation to mosquito cells deeply modified the relative abundance of each virus (Fig 7). By two viral passages, the frequency of CHIKV-LR changed from 90% to less than 10% (Fig 7A), while the frequency of the chimeric virus increased from 10% to 80%. In addition, new viruses with 3’UTRs of intermediate length arose in cell culture with a frequency of 20%. Similar results were obtained when CHIKV-Cbn was mixed with the chimeric virus carrying the 3’UTR from CHIKV-LR in a 1:9 ratio (Fig 7B). The parental CHIKV-Cbn completely displaced the Cbn/3’LR after two passages and viral variants with 3’UTRs of intermediate lengths appeared with a frequency of about 20%.

To explore whether new viral variants were the result of RNA recombination events between the 3’UTRs of different re-emergent CHIKV lineages, we compared the 3’UTR of individual sequences with those of parental CHIKV-Cbn and CHIKV-LR (S5 Fig). As represented in Fig 7C, the 3’ sequences of almost 66% of the new variants were generated by deletions of DR copies from the 3’Cbn, indicating that recombination had occurred between two RNA molecules from the same lineage. In support of a template switch mechanism for recombination, 33% of the new variants contained chimeric 3’UTRs, with a 5’ portion corresponding to the 3’LR and a 3’ portion corresponding to the 3’Cbn. As a consequence of recombination, the 3’LR gains an extra DR3 copy generating a longer 3’UTR.

Altogether, our results demonstrate that (i) viruses that have duplicated DR elements at the 3’UTR rapidly outcompete those with less copies of DRs in mosquito cells, (ii) displacement of variants with shorter 3’UTRs can be unequivocally attributed to the impact of the 3’UTR region in viral fitness and is independent of the backbone, and (iii) RNA recombination by copy-choice mechanism occurs when a host cell is infected by two or more molecules of CHIKV. Template switching of viral polymerase takes place either between viruses from the same or from different lineages.

## Discussion

**Fig 8:**
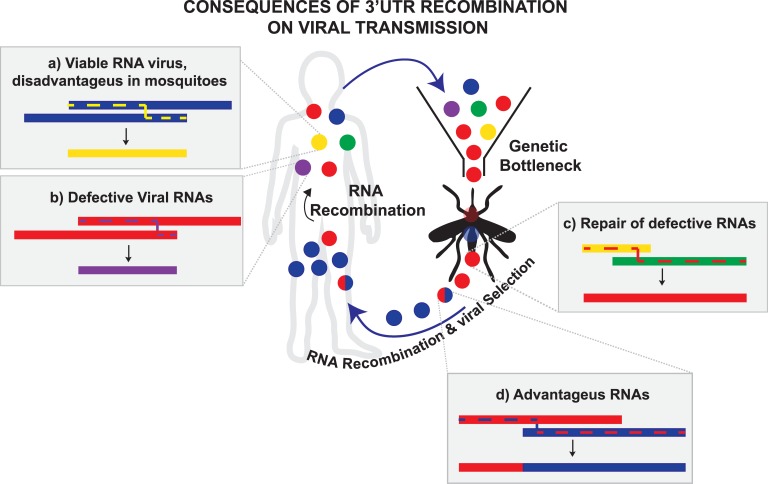
Role of RNA recombination and natural selection on the transmission cycle of CHIKV. Schematic representation of CHIKV populations during transmission. Blue and red circles represent wild type viruses with 3’UTRs of different lengths. Yellow and green circles represent deletion variants that disfavor viral replication in mosquitoes. Purple circles represent deletion variants that result in the formation of defective interfering particles. Red/blue circles are chimeric products from recombination between homologous strands (i.e. corresponding to different lineages). Recombination events that generate viral variants are schematized inside the grey boxes. The predicted routes of viral polymerase are indicated with dashed lines. In mammalian cells, deletion variants are generated by RNA recombination and positively selected due to redundancy of DRs. Within mosquitoes, the variants with higher fitness, which are less represented in the population, might not pass through genetic bottlenecks. Under this scenario, RNA recombination might act as an efficient mechanism to generate more competent viral variants that are positively selected rescuing viral replication.

Here, we followed the evolution of CHIKV 3’UTR during viral transmission with the aim of deciphering the significance of sequence repetitions for virus adaptation. Our experiments reveal that populations of CHIKV are greatly dynamic and the copy number of sequence repetitions at the 3’UTR varies continuously when the virus shuttles from mosquito to mammalian cells. Indeed, sequence repetitions act as functional blocks that are gained or lost during cycling to accelerate viral adaptability. Interestingly, duplication of complex RNA structures is a common feature of the 3’UTR among other RNA viruses that must cycle between hosts [26,37]. However, we have only recently obtained experimental insights into the function of duplicated structures for DENV, showing that duplication of a 3’ structure is beneficial in mammalian cells [27]. Here we uncovered the requirements and the underlying mechanism that maintain sequence duplications during CHIKV host cycling. Although there are evidences that support that sequence duplications at the 3’UTR provide RNA viruses with the means to replicate in hosts with conflicting demands, our results highlight the differences related to viral biology. We propose a model where RNA recombination and natural selection act concertedly to shape the composition of CHIKV populations (Fig 8). One example is the outcome of CHIKV-Cbn experimental adaptation to mammalian cells (see experiment II in Fig 3), where the same deletion variant was isolated with high frequency. This can be explained by an early single recombination event followed by positive selection during passaging in cell culture.

RNA recombination is driven by blocks of repeated sequences and is the main mechanism underlying genome plasticity at the 3’UTR in both mosquito and mammalian hosts. While positive selection favors the replication of advantageous viral variants, RNA-recombination does not always produce beneficial rearrangements of the viral genome. In fact, RNA recombination yields both viable viruses and non-replicative viral RNAs that are packaged into defective interfering particles [38] [see Fig 8A) and 8B)]. The Cbn strain missing DR3a and DR3b copies isolated in BHK cells is an example of those non-replicative RNAs. In turn, viable viruses are subjected to positive selection processes in each host, according to specific requirements for viral replication. Direct repeats at the 3’UTR are under different host-selective pressures. While they are advantageous for replication in mosquito cells, they are rather redundant in mammalian cells. These conflicting requirements explain why viral variants enriched in DR copies are positively selected in mosquito cells, and then cleared out when the virus switches to mammalian cells. However, the cause for selection of viral variants with disparate 3’UTRs in different hosts still remains unclear. In the case of DENV, we discovered that host adaptation modulates the species of non-coding RNAs derived from the 3’UTR (known as sfRNA) that are accumulated in infected cells [28]. A number of studies have revealed important functions of sfRNAs, including the evasion of immune responses [39,40]. Evidence so far suggests that during CHIKV infection no sfRNAs are produced [41]. In turn, alphavirus 3’UTR in the context of the complete viral genome appears to be the target for antiviral responses in mammalian cells: (i) CHIKV 3’UTR is specifically recognized by RIG-I receptors, that sense viral infection to initiate and modulate antiviral immunity [42] and (ii) Eastern equine encephalitis virus (EEEV) contains at its 3’UTR a binding site for a vertebrate-cell specific miRNA that restricts viral replication [43]. Therefore, it is tempting to speculate that loss of DR copies at the 3’UTR serves as a mechanism to evade host antiviral response in mammalian cells. Future work is required to elucidate whether immune escape is the driving force for CHIKV 3’UTR evolution.

It is well known that the repertoire of viral variants that appear in mammals experience important population bottlenecks when they pass to insect vectors [23–25]. Multifactorial circumstances (i.e. vector genetic variations, composition of viral populations, and specific mosquito/virus interactions) could influence the bottleneck size and viral genetic diversity. Following this line of thought, we explain the evolution of CHIKV during host switch under two possible scenarios: “tight” or “wide” genetic bottlenecks. In light of our experiments, we propose that under “wide” genetic bottlenecks the mammalian-adapted population undergoes a purifying selection to remove the variants with lower fitness in mosquitoes. On the other hand, “tight” genetic bottlenecks reduce genetic diversity. Variants with higher fitness in mosquitoes are underrepresented in the mammalian-adapted population and may not go through the bottleneck, posing a constraint to viral replication. In this scenario, RNA recombination can generate new viral variants that are positively selected in mosquitoes, working as an efficient strategy to rescue CHIKV from “tight” genetic bottlenecks or even providing an advantage for viral replication [Fig 8(C) and 8(D)].

Some parallelism can be established between in vitro and in vivo models of CHIKV replication. CHIKV propagates and grows to high titers in mosquito and mammalian cells. In nature, CHIKV quickly disseminates and viral genome reaches up to 10^7^−10^9^ copies per milliliter of blood or per infected mosquito, as early as 2 days after infection. Comparable effects of 3’UTR deletions on CHIKV replication were also observed in C6/36 cells and mosquitoes (Figs 5A,6A and Fig 6B). Overall, our study confirms previous reports showing the requirement for repeated DR elements for efficient replication in mosquito [17,21,22]. Notably, contrarily to deletion of the two DR3 repeats in the Asian lineage (21), we found that a similar deletion in the Caribbean lineage of CHIKV (see Fig 3D) did not produce infectious viruses after transfection into cultured cells, suggesting that some requirements for viral replication might be lineage specific.

The ability to switch templates has been described for several cellular and viral, DNA and RNA dependent polymerases [44–49]. For plus-strand RNA viruses, RNA recombination was reported more than 50 years ago for poliovirus [50], and later for aphthoviruses [51], coronaviruses [52], plant bromoviruses [53], flaviviruses [54] and other members of the alphaviruses genus [55]. Products from RNA recombination are proposed to impact on viral evolution and adaptation [56–58]. A strong evidence of alphavirus recombination is the emergence of Western equine encephalitis virus (WEEV), which arose from a recombination event between Eastern equine encephalitis virus (EEEV) and a Sindbis-like virus. Also, RNA recombination at the 3’UTR of Sindbis virus (SINV) has already been reported, where the conserved sequence element CSE along with the poly (A) tail have been proposed to play a role. Contrarily to our findings with CHIKV, RNA recombination of SINV RNA is not dependent on sequence homology. Instead, cryptic polymerase recognition motifs promote non-homologous template switching between viral and non-viral templates [59,60]. It appears that, although stimulated by different RNA determinants, RNA recombination is a founding mechanism that generates diversity at alphaviruses 3’UTR.

For many RNA polymerases, biochemical studies have been performed in order to explore template requirements for homologous RNA recombination, without the bias generated by selection of RNAs that are more competent for replication [54,61]. The strength of base pairing regions between the donor and the acceptor RNA strands is believed to modulate genome propensity for this process. In this regard, not all viral variants have the potential to generate diversity through RNA recombination. For instance, previous studies show that CHIKV-La Reunion carrying the deletion of both DR1 and both DR2 copies does not introduce changes at the 3’UTR during adaptation. Instead, it acquires compensatory mutations in protein coding regions, so as to improve viral fitness in mosquito cells [22]. It seems reasonable that a minimum number of DR copies should be maintained to drive RNA recombination between homologous strands. Here, although bearing certain sequence variations, we have detected recombination events when viruses containing the 3’UTR from different CHIKV lineages coexist in the same cell. During outbreaks, coinfections of mosquitoes and patients with different arboviruses have been extensively documented, supporting the notion that simultaneous exposure of mosquitoes to multiple arboviruses during one feeding episode is highly frequent [[62–65], for review see [66]]. Given the fact that ECSA and Asian derived lineages co-circulate in the same geographic areas [17,31–34], we propose that RNA recombination may occur in mosquitoes that are simultaneously infected with different CHIKV lineages, resulting in the emergence of new viral variants. The ability of viral genomes to recombine within mosquitoes could clearly have implications on CHIKV epidemiology and evolution.

Evidence for RNA recombination during natural evolution of CHIKV can be also identified in nature. For instance, the CHIKV-Cbn may have gained its extra DR copy by this molecular mechanism. Overall, this study provides clear evidence that RNA copy-choice recombination between strands from the same or different CHIKV lineages is responsible for genome diversification. In mammalian hosts, new viral variants get rid of redundant DRs; while in mosquito vectors, they might allow CHIKV to overcome “tight” genetic bottlenecks.

Uncovering the mechanisms that govern genetic diversity will certainly contribute to understand the unique potential of CHIKV to adapt to new environments.

## Materials and methods

### Sequence alignment, repeated pattern identification and structural analysis

CHIKV 3’UTR sequences were downloaded from GenBank database and aligned using ClustalW2 and MAFFTs programs. Strains with incomplete 3’UTRs were excluded from the analysis. Internal duplications were identified using LALIGN, the pairbase sequence alignment tool available at ClustalW2 site. Structure of the 3’UTR was predicted using RNAalifold software.

### Construction of recombinant CHIKVs

To facilitate mutations within the 3’UTR of CHIKV-Cbn [17] and CHIKV-LR [67] infectious clones, a unique *SacI* restriction site was introduced downstream of the stop codon of the viral structural proteins. To this end, the PCR product generated with primers sense 94 and antisense 92, and the product of a second PCR generated with primers sense 93 and antisense 95, were fused by overlapping PCR. The *Xho*I- *Not*I fragment of CHIKV-Cbn or CHIKV-LR was replaced by the *Xho*I- *Not*I fragment of the overlapping PCR products to generate CHIKV-Cbn *Sac*I or CHIKV-LR *Sac*I, respectively. A 3’UTR cloning cassette between unique *SacI* and *NotI* restriction sites allowed us to exchange the wild type sequences by mutant 3’UTRs, as described in S1 Table. Nucleotide sequences of primers used for PCRs are listed in S2 Table.

The chimeric viruses were obtained digesting the infectious clones with *SacI* and *NotI* and introducing the products in the alternate lineage. The DNAs of the recombinant constructs were linearized by digestion with *NotI* enzyme and used as templates for transcription by SP6 polymerase in the presence of GpppG cap structure analog, using the mMessage mMachine transcription kit (Thermo Fisher) according to manufacturer’s instructions. The RNAs were gel-quantified and used for transfection in cell culture.

### Cells, viral transfections and infections

Mammalian BHK-21 cells (*Mesocricetus auratus hamster kidney*, ATCC, CCL-10) were grown at 37°C in MEM alpha medium. A primary line of human skin fibroblasts was established at the Instituto de Medicina Translacional e Ingeniería Biomédica [CONICET, Hospital Italiano de Buenos Aires,[68]] and grown at 37°C in D-MEM medium. Mosquito C6/36 (*Aedes albopictus*, ATCC, CRL-1660) cells were grown at 28°C in Leibovitz L-15 medium. For RNA transfections, cell lines were grown to 60–70% confluence and transfected in 24-well plates using Lipofectamine 2000 (Invitrogen) following manufacturer’s instructions. Viral stocks were obtained by transfection of 500 ng of in vitro transcribed viral RNA and harvested from the cell culture supernatant at different times post-transfection. Viruses were quantified by plaque assays. To this end, 10^5^ Vero cells (*Cercopithecus aethiops kidney*, ATCC CCL-81) per well were seeded in 24-well plates and allowed to attach overnight. Viral stocks were serially diluted and 0.1 ml was added to the cells and incubated for 1 h. Then, 1 ml of overlay (1X D-MEM medium, 2% fetal bovine serum, 100 U of penicillin/ml and 0.4% methylcellulose) was added to each well. Cells were fixed 3 days post-infection with 10% formaldehyde and stained with crystal violet.

### Growth curves

Sub confluent BHK-21,C6/36 cells and human fibroblasts in a in a six-well plate were infected with equal amounts of CHIKV-Cbn, CHIKV-LR or mutant viruses recovered from C6/36 cells. A multiplicity of infection (MOI) of 0.1 in 500 μl of PBS was used. One hour post-infection, the cells were washed 5 times with PBS and 2 ml of growth media were added. At each time point, cell supernatants were collected and frozen at -70°C. For virus quantification, supernatants were serially diluted and plaque assay was performed on Vero cells as described above.

### Immunofluorescence assay

BHK-21 and C6/36 cells seeded into a 24-well plate on a 1 cm^2^ coverslip were transfected with CHIKV-Cbn, CHIKV-LR, or mutant RNAs, and immunofluorescence assay (IF) was performed at different times post-infection. At each time point cells were fixed in methanol and stained with a 1:1000 dilution of mouse anti-CHIKV monoclonal antibody CHK-152 [69] in PBS to detect viral antigens. Alexa Fluor 488 goat anti-mouse (Molecular Probes) was used as secondary antibody at 1:1000 dilutions. Estimation of the number of positive cells and calculation of relative percentages of infection was performed with ImageJ, using 5 images for each experimental condition.

### Experimental host adaptation and sequencing

Two independent RNA transfections were performed for each adaptation experiment. In vitro transcribed CHIKV-LR or CHIKV-Cbn RNAs were transfected into BHK-21 or C6/36 cell lines. Viruses were harvested at 2 days and five successive infections were performed in the same cell line using a MOI of 1. Viral RNAs were Trizol-extracted from culture supernatants and used for RT-PCR reactions with primer 115, complementary to the poly(A) tail plus the last 7 nucleotides of CHIKV genomes. PCR reactions were carried out using primers 115 and 116. Products were ligated into pGEM-T Easy vector (Promega) and used to transform XL1-Blue bacteria. For each passage 5 population, 26 individual clones were analyzed. The length of individual viral 3’UTRs was estimated by resolving the product from the *EcoR*I plasmid digestion in 2% agarose gels. Individual plasmid clones were sequenced by Sanger method using M13R and M13F primers.

### Assessment of recombination after co-transfection of WT and marked RNAs

A mixture of in vitro transcribed Cbn-WT and Cbn-*Sac*I/*Nhe*I RNAs was transfected into BHK or C6/36 cells. Two independent RNA transfections were performed in each case. As before, viral RNAs were Trizol-extracted from culture supernatants at day 3 and used for RT reactions with primer 115. PCR reactions were carried out using primers 115 and 123 and cloned into pGEM-T Easy vector. For each experiment, 14 individual clones were analyzed. To assess the presence of *Sac*I and/or *Nhe*I restriction sites in individual clones, 3’UTRs were amplified by PCR reactions using primers M13F and M13R (see S3 Fig). Then, PCR products were digested with *Sac*I or *Nhe*I restriction enzymes and analyzed by electrophoresis on a 2% agarose gel. Because the M13F and M13R primers anneal at different distances from the insertion site in pGEM-T Easy, the size of the bands generated by digestion depends on directionality of the cloned insert. For confirmation, 3’UTRs were also sequenced by Sanger method.

### Co-transfection of WT and chimeric RNAs

Two independent RNA transfections were performed for each parental/chimeric mix. Viral RNAs of CHIKV-LR, CHIKV LR/3’Cbn, CHIKV-Cbn and CHIKV-Cbn/3’LR were obtained by in vitro transcription and quantified. Based on the RNA concentration, mixes of CHIKV-LR and CHIKV LR/3’Cbn (9:1), or CHIKV-Cbn and CHIKV-Cbn/3’LR (1:9) were used for transfections into C6/36 cells. Viruses recovered from supernatants were used to re-infect fresh cells. As described above, RNA was extracted, used as template for RT-PCR reaction and ligated into pGEM-T Easy vector. For each experiment, 26 Individual clones were sequenced by Sanger method and ratio of viral variants was calculated.

### Mosquitoes rearing, feeding and titration

Laboratory colonies of *A*. *albopictus* were established from field collections in 2011 in Phu Hoa, Ben Cat District, Binh Duong Province, Vietnam. All the experiments were performed within 19 generations of laboratory colonization. The insectary conditions for mosquito maintenance were 28°C, 70% relative humidity, and a 12-h light and 12-h dark cycle. Adults were maintained with permanent access to 10% sucrose solution. Adult females were offered commercial rabbit blood (BCL) twice a week through a membrane feeding system (Hemotek Ltd.). 6–8 days old female mosquitoes were fed with 10^5^ PFU of CHIKV diluted in preached human blood (iCareB platform, Institut Pasteur). Mosquitoes were offered the infectious or control blood meal for 30 min through a membrane feeding system (Hemotek Ltd) set at 37°C with a piece of desalted pig intestine as the membrane. Following the blood meal, fully engorged females were selected and incubated for 2 and 8 days at 28°C, 70% relative humidity and under a 12 h light: 12 h dark cycle with permanent access to 10% sucrose. Viral titers in individual mosquitoes were determined by plaque assay. Also, total RNA was extracted with TriZol reagent and the composition of the 3’UTR in the population was analyzed as described above.

### Human blood and ethics statement

Human blood used to feed mosquitoes was obtained from healthy volunteer donors. Healthy donor recruitment was organized by the local investigator assessment using medical history, laboratory results and clinical examinations. Biological samples were supplied through participation of healthy volunteers at the ICAReB biobanking platform (BB-0033-00062/ICAReB platform/Institut Pasteur, Paris/BBMRI AO203/[BIORESOURCE]) of the Institut Pasteur to the CoSImmGen and Diagmicoll protocols which have been approved by the French Ethical Committee (CPP) Ile-de-France I. The Diagmicoll protocol was declared to the French Research Ministry under the reference: DC 2008–68 COL 1.

## Supporting information

S1 Fig Nucleotide sequences of CHIKV 3’UTRs during adaptation to mosquito cells. Alignment of nucleotide sequences corresponding to the 3’UTR during adaptation experiments to C6/36 cells from Fig 3 is shown. Input Cbn-WT sequence is presented as reference. Numbers on the left correspond to those of the clones schematized in Fig 3C (left). Nucleotide changes are indicated in orange. Position 1 refers to the first position after the translation stop codon.
(EPS)

S2 Fig Nucleotide sequences of CHIKV 3’UTRs during adaptation to mammalian cells. Alignment of nucleotide sequences corresponding to the 3’UTR during adaptation experiments to BHK cells from Fig 3 is shown. Input Cbn-WT sequence is presented as reference. Numbers on the left correspond to those of the clones schematized in Fig 3C (right). Nucleotide changes are indicated in red. Position 1 refers to the first position after the translation stop codon.
(EPS)

S3 Fig Strategy for detection of *Sac*I and *Nhe*I restriction sites in individual clones. Individual clones of pGEM-T Easy containing CHIKV 3’UTRs were used as template for PCR reactions with M13F and M13R primers. The 3’UTR insert and pGEM-T Easy vector are represented in red and grey, respectively. Then, amplified fragments were digested with *Sac*I or *Nhe*I restriction enzymes. Because M13F and M13R primers anneal at different distances from the T-overhangs in pGEM-T Easy, directionality of the insert impacts on the band sizes generated after digestion of the PCR products. The expected sizes of the bands are indicated for an insert in the two possible orientations of DNA ligation (A or B).
(EPS)

S4 Fig Replication kinetics of CHIKV-Cbn WT and Δ(1+2)abb’ in human fibroblasts. Top, schematic representation of the 3’UTR corresponding to the CHIKV-Cbn wild type (Cbn WT) and the recombinant virus lacking three copies of DR (1+2) [Cbn Δ(1+2)abb’]. DR copies are represented with colored blocks and DR deletions are indicated with light grey blocks. Bottom, comparative growth kinetics (left) and immunofluorescence at 2 days post-infection (right) of Cbn WT and Cbn Δ(1+2)abb’in human fibroblasts. Error bars represent standard deviations from the mean, n = 3.
(EPS)

S5 Fig Nucleotide sequences of the chimeric 3’UTRs produced by a copy-choice mechanism. Alignment of nucleotide sequences of recombinants generated by template switch from Fig 7 is shown. Input LR-WT and Cbn-WT are presented as reference. Lineage-specific nucleotides from LR-WT and Cbn-WT are shown in red and blue, respectively. Sequence labels matches the numbering of individual clones 8 and 16 in Fig 7. Changes detected in chimeric RNAs are indicated with the respective colors. Sites of template switching are marked with orange arrows. Position 1 refers to the first position after the translation stop codon.
(EPS)

S1 Table Strategy used to design mutant RNAs. (DOCX)

S2 Table Sequence of primers used throughout the manuscript. (DOCX)
