# Supplementary material for: RNA recombination at Chikungunya virus 3'UTR as an evolutionary mechanism that provides adaptability
Source: PLoS Pathog. 2019 Apr 15;15(4):e1007706. doi: 10.1371/journal.ppat.1007706 (PMC6502353; doi:10.1371/journal.ppat.1007706)
Supplement: S1 Table — (DOCX) [file ppat.1007706.s006.docx]

| Mutant | Strategy | DNA template for PCR |
| --- | --- | --- |
| LR *Sac*I | Overlapping PCR using primers 92-94 and 93-95 | CHIKV-LR |
| Cbn *Sac*I | Overlapping PCR using primers 92-94 and 93-95 | CHIKV-Cbn |
| Cbn Δ(1+2)a | PCR primers 118 and 92 | CHIKV-Cbn *SacI* |
| Cbn Δ(1+2)ab | PCR primers 118 and 92 | CHIKV-Cbn *SacI* |
| Cbn Δ(1+2)abb’ | PCR primers 96 and 92 | CHIKV-Cbn *SacI* |
| Cbn Δ3a | Overlapping PCR using primers 128-92 and 129-94 | CHIKV-Cbn *SacI* |
| LR Δ1abΔ2ab | PCR primers 96 and 92 | CHIKV-LR *Sac*I |
| Cbn *Sac*I/*Nhe*I | Overlapping PCR using primers 124-92 and 125-94 | CHIKV-Cbn *SacI* |
