# Supplementary material for: RNA recombination at Chikungunya virus 3'UTR as an evolutionary mechanism that provides adaptability
Source: PLoS Pathog. 2019 Apr 15;15(4):e1007706. doi: 10.1371/journal.ppat.1007706 (PMC6502353; doi:10.1371/journal.ppat.1007706)
Supplement: S2 Table — (DOCX) [file ppat.1007706.s007.docx]

| 92 | 5’-CGAAACAAGCGCTCATGAGC-3’ |
| --- | --- |
| 93 | 5’-GACTGAAGGGCTCGAGGTCA-3’ |
| 94 | 5’-TCAGCAGGCACTAAgagCTcGACAATTAAGTA-3’ |
| 95 | 5’-TACTTAATTGTCGAGCTCTTAGTGCCTGCTGA-3’ |
| 96 | 5’-GCACTAAgagCTcGTTCAAAGGGCTATAaaacccc-3’ |
| 98 | 5’-TAACATCTCCTACGTGCCCTTTGAACTACTTCTG-3’ |
| 115 | 5’-TTTTTTTTTTTTTTTTTTTGAAATAT-3’ |
| 116 | 5’-CTAATCGTGGTGCTATGC-3’ |
| 118 | 5’-TAAGAGCTCTACATAAATAGGTATAC-3’ |
| 123 | 5’-ACTAACGCCGTCACTATTCG-3’ |
| 124 | 5’-ACCCATAGGGACGCTAGCAGATGTTATTTTG-3’ |
| 125 | 5’- CAAAATAACATCTGCTAGCGTCCCTATGGGT-3’ |
| 128 | 5’-AGAAGTAGTTCAAAGGGAGATGTTCAAAGTGAC-3’ |
| 129 | 5’- GTCACTTTGAACATCTCCCTTTGAACTACTTCT-3’ |
| M13R | 5’-CAGGAAACAGCTATGAC-3’ |
| M13F | 5’-GTTTTCCCAGTCACGAC-3’ |
